# Notice of “Answer to Dr. Ruppaner”

**Published:** 1870-10

**Authors:** Lewis A. Sayre

**Affiliations:** Professor of Orthopædic Surgery, etc., etc.


*Answer to Dr. Ruppaner;* with a Review and Criticism on his recent report of a Case of Laryngo-Tracheotomy, including all the attested facts regarding the recent Partridge Poisoning Case at the Fifth Avenue Hotel. By LEWIS A. SAYRE, M.D., Professor of Orthopædic Surgery, etc., etc.,

Is the somewhat voluminous title of a pamphlet just received, with the endorsement "please notice," which we shall do, with all due courtesy to its author, by deprecating the publication of differences purely private and personal in their nature, such as the subject matter of the pamphlet before us. Personal quarrels generally possess very little interest for the public, and especially is this the case when the belligerents are physicians, "Doctors' differences" having passed into a proverb.

We have yet to learn a single instance of benefit to a professional reputation, by enlisting the public as partizans for its defense when assailed, while the profession at large are repeatedly made painfully conscious of detriment sustained in such contests. The subjects of difference treated in the pamphlet under discussion, could have interested none outside of the circle of personal acquaintance of the parties concerned, and these have doubtless long since formed decided opinions, which will undergo no modification by further evidence or discussion; while the profession at large, not having all the evidence upon both sides of the question, cannot possibly form a rational opinion. From such premises and from such only are we willing to form an opinion, but not even then to express one upon a purely private matter.

